# Deficiency of functional mannose-binding lectin is not associated with infections in patients with systemic lupus erythematosus

**DOI:** 10.1186/ar2095

**Published:** 2006-12-13

**Authors:** Irene EM Bultink, Dörte Hamann, Marc A Seelen, Margreet H Hart, Ben AC Dijkmans, Mohamed R Daha, Alexandre E Voskuyl

**Affiliations:** 1 Department of Rheumatology, VU University Medical Center, Postbox 7057, Amsterdam 1007 MB, The Netherlands; 2 Slotervaart Hospital, Louwesweg 6, Amsterdam 1066 EC, The Netherlands; 3 Jan van Breemen Institute, Dr J. van Breemenstraat 2, Amsterdam 1056 AB, The Netherlands; 4 Sanquin Research at CLB, Plesmanlaan 125, Amsterdam 1066 CX, The Netherlands; 5 Department of Nephrology, Leiden University Medical Center, Postbox 9600, Leiden 2300 RC, The Netherlands

## Abstract

Infection imposes a serious burden on patients with systemic lupus erythematosus (SLE). The increased infection rate in SLE patients has been attributed in part to defects of immune defence. Recently, the lectin pathway of complement activation has also been suggested to play a role in the occurrence of infections in SLE. In previous studies, SLE patients homozygous for mannose-binding lectin (MBL) variant alleles were at an increased risk of acquiring serious infections in comparison with patients who were heterozygous or homozygous for the normal allele. This association suggests a correlation between functional MBL level and occurrence of infections in SLE patients. We therefore investigated the biological activity of MBL and its relationship with the occurrence of infections in patients with SLE. Demographic and clinical data were collected in 103 patients with SLE. Functional MBL serum levels and MBL-induced C4 deposition were measured by enzyme-linked immunosorbent assay using mannan as coat and an MBL- or C4b-specific monoclonal antibody. The complete MBL-dependent pathway activity was determined by using an assay that measures the complete MBL pathway activity in serum, starting with binding of MBL to mannan, and was detected with a specific monoclonal antibody against C5b-9. Charts were systematically reviewed to obtain information on documented infections since diagnosis of SLE. Major infections were defined as infections requiring hospital admission and intravenous administration of antibiotics. In total, 115 infections since diagnosis of lupus, including 42 major infections, were documented in the 103 SLE patients (mean age 41 ± 13 years, mean disease duration 7 ± 4 years). The percentage of SLE patients with severe MBL deficiency was similar to that in 100 healthy controls: 13% versus 14%, respectively. Although deposition of C4 to mannan and MBL pathway activity were reduced in 21% and 43% of 103 SLE patients, respectively, neither functional MBL serum levels nor MBL pathway activity was associated with infections or major infections in regression analyses. In conclusion, SLE patients frequently suffer from infections, but deficiency of functional MBL does not confer additional risk.

## Introduction

Infections are an important cause of morbidity and mortality in patients with systemic lupus erythematosus (SLE). Infectious complications occur in 25% to 45% of SLE patients in case series [1,2], and infection as cause of death has been reported in up to 50% of patients with SLE [3,4]. The increased infection rate in patients with SLE has been attributed in part to defects in the complement system, which has an important role in host defence against microorganisms [3].

Genetic deficiencies of early components of the classical pathway of complement activation are strongly associated with the development of SLE [5]. In particular, deficiency of C1q is a major predisposing risk factor for SLE. C1q plays a role in the recognition and clearance of apoptotic material [6] and binds predominantly to antibodies and protein structures on bacteria and viruses, resulting in complement activation.

More recently, the lectin pathway of complement activation has also been suggested to play a role in the pathogenesis of SLE [7] and in the occurrence of infections in SLE [8-10]. Mannose-binding lectin (MBL) is a serum protein with characteristics very similar to those of C1q [11]. MBL may activate complement through the lectin pathway by interacting with MBL-associated serine proteases (MASPs). Furthermore, MBL can directly opsonise pathogens and enhance the activity of phagocytes [12].

Homozygosity for variant MBL alleles is probably a minor risk factor for the presence of SLE, as shown by a recent meta-analysis of all available case-control studies. In that study, a significant association between MBL codon 54 variant B and SLE was demonstrated [13]. Genetic and phenotypic deficiency in producing MBL has been associated with recurrent or serious infections, mainly in children [14] and in immune-compromised individuals [15].

In three previous studies, SLE patients homozygous for MBL variant alleles were at an increased risk for serious infections compared with patients who were heterozygous or homozygous for the normal allele [8-10]. This association suggests a correlation between functional MBL level and occurrence of infections in these patients. However, the association between rates of infection and functional MBL serum levels has not been studied yet.

Genotype predicts MBL serum concentration reasonably well at the population level. However, in individuals, even full genotypic characterisation is insufficient to predict functional MBL serum levels [16,17]. Moreover, functional MBL serum levels not only are determined by MBL genotype and promoter polymorphisms but also are influenced by MASP activity and serum levels of other complement factors. Assays are available to test these separate influences [18]. The capacity of highly oligomerised MBL to bind to microorganisms can be tested *in vitro *by incubating serum on mannan-coated plates and subsequently detecting bound MBL with an MBL-specific monoclonal antibody. This assay is dependent only on the amount of functional MBL protein. Activity of the MBL/MASP complex is determined by performing the incubation on mannan-coated plates at 37°C and subsequently detecting C4 deposition on the surface. Functional MBL serum levels and C4 deposition are highly correlated except in cases of MASP deficiency. Functional activity of the entire MBL pathway can be measured starting with binding of MBL to mannan and detection of the membrane-attack complex C5–9. This assay is sensitive to defects in all components of the MBL pathway. Following the above methodology, we measured functional MBL activity in our clinic population of patients with SLE and related this to past infectious events.

## Materials and methods

### Patients and data collection

One hundred and three patients fulfilling the revised criteria for the classification of SLE [19] were included in the study. All patients were regular outpatients of the rheumatology clinic of the VU University Medical Center, the Jan van Breemen Institute, or the Slotervaart Hospital. These clinics provide primary through tertiary care for patients with SLE in Amsterdam, The Netherlands. The local ethics committee approved the study. All patients provided informed consent for their participation. Demographic, patient, and disease characteristics were systematically recorded by interview, self-reported questionnaire, chart review, and clinical examination performed by one rheumatologist (IEMB).

### Definition of infections

Infections were included in the analysis if they met the following criteria: documentation in medical records (patient charts and microbiology laboratory reports), occurring since diagnosis of lupus, and confirmed both by clinical findings and positive cultures. If bacterial isolates were not available (for example, in some cases of pneumonia, sinusitis, and otitis), infection was diagnosed by clinical manifestations in combination with radiographic findings and response to antibiotic treatment. Lower urinary tract infections were excluded because of potential under-reporting of this infection. Viral infections were included if clinical symptoms of a viral infection were present in combination with positive culture or confirmed by high titres of antibodies for virus-specific antigens. Herpes zoster infections were included if typical dermatomal vesicular cutaneous lesions had occurred. Major infections were defined as infections for which hospital admission and intravenous antibiotic treatment had been required. Opportunistic infections were defined as infections with pathogens that are uncommon in the non-compromised host.

### Laboratory investigations

#### Assessment of functional MBL serum levels and C4 concentration

Fifty micrograms per millilitre of mannan (Sigma-Aldrich, St. Louis, MO, USA) was coated overnight in coating buffer (0.1 M carbonate, pH 9.6) on a Nunc Maxisorp microtitre plate (Invitrogen, Breda, The Netherlands). All incubations were performed in a volume of 100 μl at room temperature. Plates were washed five times with water (all other washes were also carried out in water), and samples were diluted 1:3, 1:9, 1:27, and 1:81 in TTG/Ca^2+ ^(20 mM Tris, 150 mM NaCl, 0.2% gelatin [wt/vol], 0.02% TWEEN-20 [wt/vol], 10 mM CaCl_2_, pH 7.4) for 1 hour. A standard curve was generated using two-fold serial dilutions of a pool of 3,000 sera obtained from blood bank donors, containing 1.5 μg/ml of MBL when compared with a standard from Dade Behring Holding GmbH (Eschborn, Germany). Plates were washed five times and incubated with 2 μg/ml of biotinylated CLB-MBL/1 in TTG/Ca^2+ ^for 1 hour. After washing five times, the plates were incubated with polymerised streptavidin-horseradish peroxidase (HRP) (Sanquin, Amsterdam, The Netherlands) diluted 1:10,000 in Tris-buffered saline/Ca^2+^/milk (20 mM Tris, 150 mM NaCl, 10 mM CaCl_2_, 2% [vol/vol] cow's milk, pH 7.4). Plates were incubated for 30 minutes and washed five times. The assay was developed with 100 μg/ml TMB (3,3',5,5'-tetramethylbenzidine) in 0.11 M sodium acetate (pH 5.5) containing 0.003% H_2_O_2 _(vol/vol). Substrate conversion was stopped by adding 100 μl of 2 M H_2_SO_4_, and absorbance was measured at 450 nm. The assay was validated by testing 100 blood bank donors using either CLB-MBL/1 as described above or HYB 131-1 (a kind gift of S. Thiel, University of Aarhus, Aarhus, Denmark), which revealed comparable results. A cutoff value of less than 0.05 μg/ml MBL corresponds to low-level-producing MBL genotypes [16]. Serum levels of C4 were measured by nephelometry (Dade Behring Holding GmbH).

#### Assessment of MBL-induced C4 deposition on mannan

Plates were coated overnight in coating buffer with 50 μg/ml of mannan. All incubations were performed in a volume of 100 μl. Serum samples were diluted 1:10, 1:30, 1:90, and 1:270 in VB/T (10 mM veronal, 150 mM NaCl, 10 mM CaCl_2_, 10 mM MgCl_2_, 0.3% bovine serum albumin [wt/vol], 0.02% TWEEN-20) and incubated for 30 minutes at 37°C. A standard curve was generated using two-fold serial dilutions of a pool of 3,000 sera obtained from blood bank donors, containing 1.5 μg/ml of MBL. Plates were washed five times with water (all other washes were also carried out in water) and incubated for 1 hour with biotinylated monoclonal antibody C4–10 [20] for measuring C4b deposition, at 0.25 μg/ml in TTG/Ca^2+^. Plates were washed five times and incubated with polymerised streptavidin-HRP as described above. Plates were washed five times and colour reaction was obtained and measured as described above. Individual results are expressed as percentage of the standard pool serum, which was set at 100%. Values below 10% point to severely decreased activity. In healthy controls, 90% of individuals with a C4 deposition activity below 10% have a functional MBL serum level of less than 0.1 μg/ml.

Alternative pathway activation is excluded by diluting the serum at least 1:10. Classical pathway activation does not influence the assay, because addition of monoclonal antibody C1q-85 [21], which inhibits activation of C1q by immune complexes, has no effect on the results. In addition, use of MBL-deficient serum has never resulted in detectable levels of C4 deposition under the described conditions.

**Figure 1:**
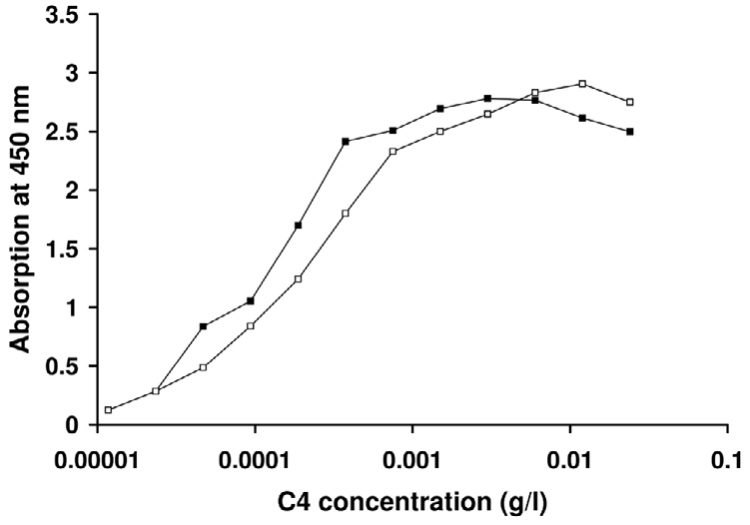
Dependence of C4 deposition assay on C4 serum concentration. C4 deposition was measured in a dilution curve of serum of two healthy donors in the presence of a constant mannose-binding lectin concentration of 200 ng/ml. C4 deposition becomes C4-dependent at a concentration below 0.001 g/l. Because sera with low C4 deposition (<10%) are evaluated at a dilution of 1:10, the minimal required C4 concentration in a tested serum will be 0.01 g/l.

C4 deposition assay results are not influenced by a low C4 concentration in serum that can be expected in patients with SLE. Serum of two healthy donors was tested in a dilution curve in the presence of a constant dilution of 200 ng/ml natural purified human MBL (a kind gift from Inga Laursen, Statens Serum Institute, Copenhagen, Denmark). C4 deposition is reduced at C4 values below 0.001 g/l. Because sera with low C4 deposition (<10%) are evaluated at a 1:10 dilution, the assay can be performed until a minimal C4 concentration of 0.01 g/l (Figure 1).

#### Antibodies

The monoclonal antibody to C4b (anti-C4–10) was generated by fusing spleen cells from mice immunised with native C4 with the mouse myeloma cell line SP2/0 [20]. CLB-MBL/1 was obtained by a fusion of spleen cells from a mouse immunised with natural MBL, purified from Cohn fraction III (Sanquin) as previously described by Kilpatrick [22]. Specificity for MBL was shown on Western blot. Affinity for the carbohydrate-binding domain was concluded from its ability to inhibit binding of MBL to mannan (data not shown).

#### Assessment of MBL pathway activation

MBL pathway activation was assessed by an enzyme-linked immunosorbent assay technique that measures the complete MBL pathway activity in serum, starting with binding of MBL to mannan, and was detected with a specific monoclonal antibody against C5b-9, as described previously [23]. To prevent contribution of the classical pathway in this assay, an antibody against C1q is added to the reaction mixture [23]. Functional activity of the MBL pathway was expressed as percentage of a standard consisting of pooled human serum set at 100%. Decreased functional activity of the MBL pathway was defined as functional activity of less than 10%.

### Statistical analyses

Differences between groups were evaluated by Mann-Whitney test; the following subgroups were considered: SLE patients with one or more major infections versus SLE patients without major infections, SLE patients with one or more major infections versus patients who experienced minor infections only, and SLE patients with one or more major infections versus SLE patients without any infections. Correlations between different assays and between assays and disease activity scores were evaluated by calculating Spearman's correlation coefficient. Variables possibly associated with the occurrence of infections and major infections were first examined by univariate tests and subsequently by multiple regression analysis. As a consequence of the low number of opportunistic infections since lupus onset in our patients, a further analysis of variables associated with the occurrence of opportunistic infections was not performed. The following variables were evaluated in relationship to the occurrence of the first major infection since lupus diagnosis by univariate analyses: disease duration, history of lupus nephritis confirmed by renal biopsy occurring before the first major infection, functional MBL serum levels, activity of the MBL pathway of complement activation, use of oral corticosteroids at the moment of first major infection, and previous use within the last 3 months before the first major infection of the following drugs: intravenous methylprednisolone, hydroxychloroquine, methotrexate, azathioprine, and (oral and/or intravenous) cyclophosphamide. To determine which factors were significantly associated with infections and major infections, the demographic, clinical, and therapy variables with a *p *value of less than 0.2 in the univariate analyses and variables with supposed clinical relevance were entered into the respective multiple regression analyses. The multiple regression models were refined by tentatively adding to the (almost) final model single variables initially not included in the model, so as to check once more whether these variables could indeed be missed. A two-sided *p *value of less than or equal to 0.05 was considered statistically significant. The software used was the Statistical Package for Social Sciences for Windows, version 13.0 (SPSS Inc., Chicago, IL, USA).

## Results

### Patient characteristics

The demographic, clinical, and therapy characteristics of the 103 patients with SLE are shown in Table 1. The majority of the patients were female Caucasians with a mean disease duration of ± 7 years. The ethnic backgrounds of the remaining 23% of patients were Asiatic (11%), negroid (8%), Mediterranean (3%), and other (1%). The disease activity and organ damage index were modest in most patients at the time of inclusion. Most patients had been treated with corticosteroids and hydroxychloroquine in the past. At the moment of inclusion, half of the patients were on treatment with both corticosteroids and hydroxychloroquine and 16% were treated with azathioprine.

### Functional MBL serum levels

The median functional MBL serum level in the patients with SLE (1.4 μg/ml, range 0.04 to 7.60 μg/ml) was comparable with that in 100 healthy laboratory workers (1.1 μg/ml, range 0.02 to 11.2 μg/ml). The prevalence of severely decreased MBL levels (<0.05 μg/ml) was similar to that of healthy laboratory workers (13% and 14%, respectively).

### Complement C4 deposition and MBL pathway activity

As shown in Table 1, the median C4 deposition in patients with SLE was 71% versus 114% in the healthy controls and was less than 10% of the activity of the standard in 21% of the patients with SLE versus 16% in healthy controls. The median functional activity of the MBL pathway in patients with SLE was 16% versus 56% in 120 healthy controls, and functional activity of the MBL pathway was less than 10% of the activity of the standard in 43% of the patients with SLE versus 28% in healthy controls (Table 1).

### Correlations between functional MBL level, biological activity of MBL, complement C4 deposition, and disease activity in patients with SLE

Both MBL pathway activation and C4 deposition were correlated to MBL serum levels (*r *= 0.75 and *r *= 0.66, respectively). In addition, C4 deposition was correlated to MBL pathway activity (*r *= 0.77, all correlations *p *= 0.0001 or less). C4 levels were poorly correlated to MBL pathway activity and C4 deposition (*r *= 0.2, *p *= 0.047 and *r *= 0.26, *p *= 0.009, respectively). MBL pathway activity was poorly correlated to C3 level (*r *= 0.2, *p *= 0.046). No consistent associations between functional MBL activity, measured by the three assays, and SLEDAI (systemic lupus erythematosus disease activity index) or ECLAM (European consensus lupus activity measurement) disease activity scores were found (data not shown).

### Infectious episodes

Fifty-one patients with SLE (50%) had suffered at least one infectious episode since lupus onset. The number of infections in a single patient ranged from zero to nine, and the mean (± standard deviation) number of infections was 1.1 (± 1.7). A total of 115 infectious episodes were documented, of which 37% were major infections. As shown in Table 2, the most common locations of infections were skin and mucosa (29%), lower respiratory tract (22%), upper respiratory tract (14%), genital (11%), gastrointestinal tract (9%), and systemic (7%). The most common infection was Herpes zoster skin infection (16%). Microorganisms were isolated in 50% of the infectious episodes: bacteria (45%), viruses (38%), and yeasts (12%).

Twenty-three patients had a total of 42 major infections. These were most commonly located in the lower respiratory tract (31%), systemic (21%), genital tract (10%), and skin and mucosa (10%). In cases in which the causal microorganism could be identified (57%), 71% proved bacterial, 17% viral, 4% fungal, and 4% a combination of viral and fungal infections. *Staphylococcus aureus *was the most frequent isolate (17%). Five patients had an opportunistic infection: oesophageal infection caused by *Candida albicans *(2), pneumonia due to *Klebsiella pneumoniae *(1), sinusitis due to *Aspergillus fumigatus *(1), and one sepsis caused by Cytomegalovirus and *C. albicans*.

### Variables associated with major infectious episodes

#### Univariate analyses

Results of univariate analyses of potential risk factors for major infections are shown in Table 3. Functional MBL serum levels and functional activity of the MBL pathway of complement activation were not different between SLE patients with major infections and those without major infections. As expected, SLE patients who had suffered at least one major infection since lupus diagnosis had a significantly longer mean (± standard deviation) disease duration in comparison with SLE patients who never had a major infection. Furthermore, the percentage of SLE patients with a previous SLE glomerulonephritis was significantly higher in SLE patients who had major infections than in SLE patients without major infections. The median serum creatinine level in SLE patients at the moment of the first major infection (74 μmol/l, range 45 to 389 μmol/l) was comparable with that in SLE patients without major infections (82 μmol/l, range 65 to 154 μmol/l). Prevalence of hydroxychloroquine use within the last 3 months before the first major infection was significantly lower than the prevalence of hydroxychloroquine use ever in SLE patients without a major infection (*p *= 0.0001).

### Multiple regression analyses

As shown in Table 4, disease duration was significantly positively associated and hydroxychloroquine use was significantly negatively associated with the occurrence of the first major infection in a multiple regression analysis that included the following variables: disease duration at follow-up; previous lupus nephritis; use of hydroxychloroquine, intravenous methylprednisolone, and intravenous cyclophosphamide within the last 3 months before the first major infection; use of each of these drugs ever in case no major infection occurred (as independent variables); and first major infection (as dependent variable). None of the other variables investigated demonstrated a significant contribution to this model.

## Discussion

Our patients with SLE frequently suffered from major infections, but we found no association between functional activity of the MBL pathway and the occurrence of infection. Strengths of our study include a high number of clinically relevant events and to our knowledge the first attempt to study functional MBL in SLE patients at risk for infection.

Functional activity of the MBL pathway in serum not only is determined by mutations in the gene encoding MBL, but also is influenced by promoter polymorphisms. Moreover, biological activity of MBL also depends on MASP activity and environmental factors can influence MBL levels in serum [16,24]. MBL is a weak acute-phase reactant [12,25], and circulating MBL levels were found to increase only 1.5- to 3-fold in non-SLE patients during an acute-phase reaction [25]. No increase was observed in individuals who were homozygous or compound heterozygous for MBL mutant alleles [26], and therefore quite stable MBL levels are present in individuals. For these reasons, functional MBL activity is thought to be a better estimate of the *in vivo *situation than nucleic acid substitutions determining genotypes when evaluating the role of the MBL pathway of complement activation in relation to the occurrence of infections in SLE.

In the present study, functional MBL activity was measured by three assays. None of these assays showed an association between deficient MBL activity and the occurrence of infections or major infections in patients with SLE. The prevalence (13%) of severe MBL deficiency in SLE patients, defined as MBL serum levels of less than 0.05 μg/ml, was similar to that in healthy laboratory workers. Functional MBL level, biological activity of MBL, and complement C4 deposition assays were significantly correlated. However, the prevalence of decreased C4 deposition (21%) and decreased MBL pathway activity in 43% of the patients with SLE was higher than the prevalence of decreased MBL serum levels in patients with SLE. As expected, both C4 and C3 levels were lower in SLE patients than in healthy controls. However, correlations between C4 or C3 and C4 deposition and MBL pathway activity were very low. MBL-induced complement activity was correlated to MBL levels in patients and healthy controls. Our findings are in contrast to a previous study in which MBL-induced C4 deposition was associated with plasma C4 levels in healthy controls and patients with SLE [27]. In that study, MBL levels were correlated to C4 deposition in healthy controls but not in patients with SLE, suggesting a high sensitivity of that assay for C4 levels. Our C4 deposition assay is dependent primarily on functional MBL plasma levels in both healthy controls and patients with SLE, and C4 is not a limiting factor until very low concentrations of 0.01 g/l in healthy controls. Although all patients with SLE had levels above the threshold, dysfunction of C4 or the presence of complement inhibitors in SLE plasma cannot be excluded, because MBL-induced complement activity is reduced in our patients. Furthermore, additional consumption of complement factors other than C4 may be relevant because we observed a higher frequency of deficient patients in the MBL pathway activity assay compared with the C4 deposition assay. Further studies are needed to investigate the role of the several complement deficiencies, dysfunction, inhibitors, and consumption.

To exclude an influence of very low C4 serum levels on both C4 deposition and MBL pathway activity assays, subanalyses of the association between laboratory variables and the occurrence of infections and major infections were performed after exclusion of SLE patients with C4 serum levels of less than or equal to 0.1 g/l (*n *= 28). In these subanalyses, C4 deposition assay and MBL pathway activity assay were not associated with infections or major infections, either in univariate and multiple regression analyses (data not shown).

**Figure 2:**
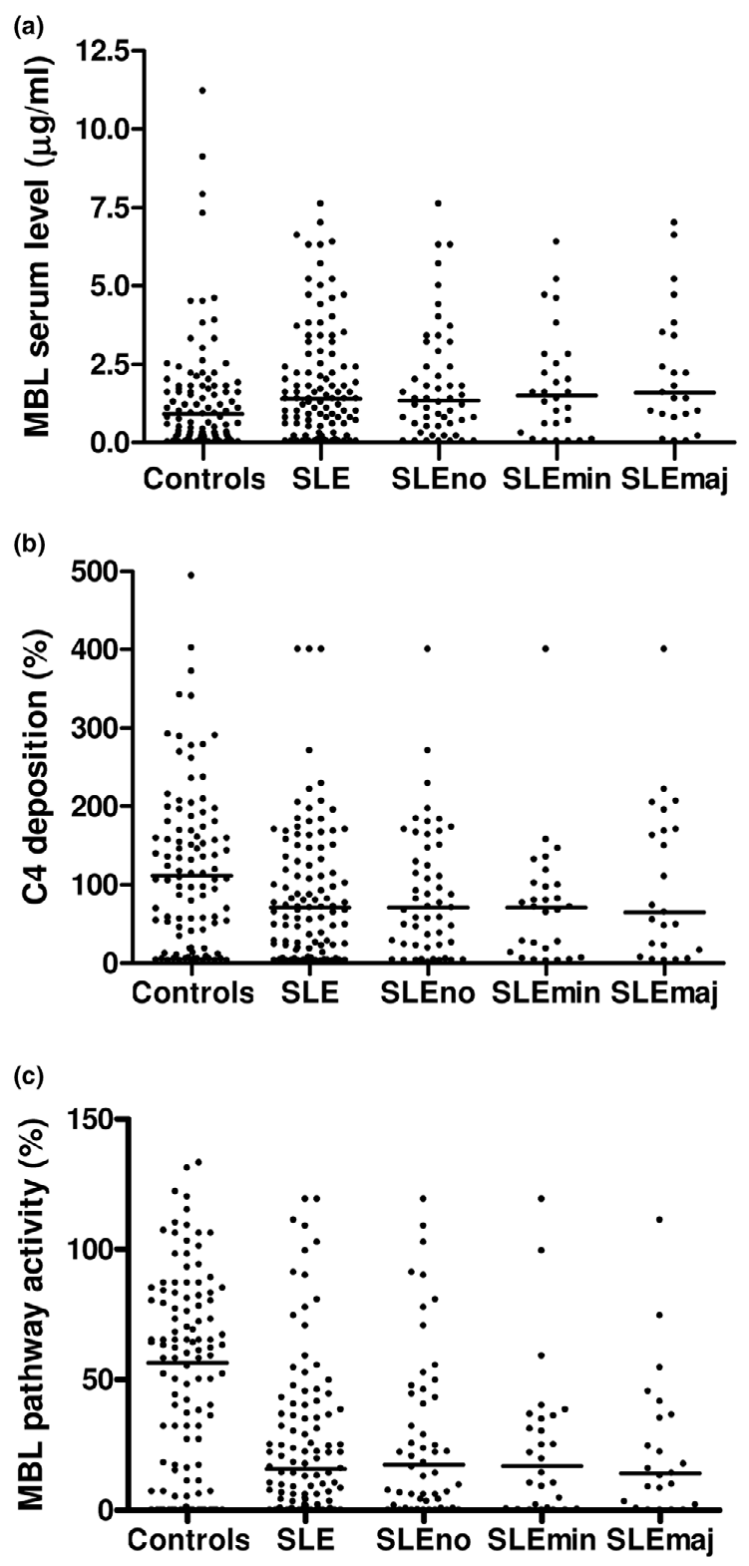
Mannose-binding lectin (MBL) serum level, C4 deposition, and MBL pathway activity. Functional MBL activity measured by three assays in all patients with systemic lupus erythematosus (SLE; *n *= 103), a subgroup of SLE patients without infections (SLEno; *n *= 52), a subgroup of patients with SLE who had minor infections only (SLEmin; *n *= 28), a subgroup of SLE patients with one or more major infections (SLEmaj; *n *= 23), and healthy controls. **(a) **MBL serum levels. **(b) **Complement C4 deposition. **(c) **MBL pathway activity. Each datapoint represents one patient. Bars show the median values. No significant differences were found between the median functional MBL activity of all patients with SLE and each of the subgroups or between the subgroups, as measured with three assays (data not shown).

When functional MBL activity with respect to the severity of infections was analysed, no significant differences were found between median values of functional MBL serum level, C4 deposition, and MBL pathway activity in three subgroups of patients: SLE patients with one or more major infections, patients with SLE who experienced minor infections only, and SLE patients without any infections (Figure 2).

Associations between MBL genotype and the occurrence of infections in patients with SLE have been reported [8-10]. In those studies, MBL serum levels were measured as well, but no association between low MBL serum levels and the occurrence of infections was demonstrated, probably because of the inability of serologic methods used to distinguish between functional and nonfunctional protein [28]. Unfortunately, no DNA is available from our patient cohort and for this reason we cannot correlate MBL genotype and risk of infections. As far as data are available, differences in clinical and epidemiological characteristics of the study patients are unlikely to be responsible for the discrepancy between the results of the present study and previous studies [8-10], except for the different racial background of the study patients in the Japanese study [10]. Another explanation for the discrepancy between the genotypic and the phenotypic data could be that unidentified linkages between mutations or polymorphisms in the MBL gene with mutations or polymorphisms in other genes might influence the genetic approach.

Despite the use of new treatment strategies to improve the clinical outcome in patients with SLE, the importance of infections as a cause of morbidity and mortality in SLE has not changed in the past decades. Defects of immune defence and treatment with corticosteroids and immunosuppressive agents are supposed to play a role in the pathogenesis of infections in SLE [3], but the mechanisms underlying the increased infection rate in SLE are not fully understood. Our study demonstrates the occurrence of at least one infectious episode since lupus diagnosis in 50% of patients with SLE, and this high infection rate is confirmed by other studies reporting infectious complications in up to 45% of SLE patients in case series [1,29]. The spectrum of infections found in our study is in line with other studies in patients with SLE, which report a broad spectrum of infections caused predominantly by community-acquired bacteria [1,29,30]. The severity of infections found in other studies in patients with SLE [1,30] is confirmed by our study, which shows a third of the infections in patients with SLE to be major infections for which hospital admission was required. Furthermore, the high incidence of Herpes zoster infection, occurring at least one time in 14% of the patients, is in line with other studies in patients with SLE [1,31].

With respect to major infections, use of immunosuppressive medication and use of corticosteroids were not associated with the occurrence of the first major infection in our patients in the best multiple logistic regression model. This finding is in line with a previous study on major infections in SLE patients in the Hopkins Lupus Cohort [32].

Renal insufficiency is a possible risk factor for infections. However, no such association was found in our study. The presence of prior SLE glomerulonephritis was not significantly associated with the occurrence of the first major infection in the best multiple logistic regression model. Moreover, the median serum creatinine level at the moment of the first major infection was similar to that in SLE patients without major infection.

Our study demonstrated a significant negative association between hydroxychloroquine use and the occurrence of major infections. This finding might be explained by the predominant use of hydroxychloroquine in the treatment of patients with mild lupus disease activity and not by the antimicrobial properties of hydroxychloroquine. Antimalarials act against pathogenic organisms that are very uncommon in Western Europe.

Limitations of the present study are the racial and socioeconomic backgrounds of the study population. As a consequence of the rather high percentage of Caucasians in the study population, the associations found in the present study may not be generalised to lupus cohorts of different racial background. However, the disease severity in our study population appears to be comparable to that in other large multi-ethnic SLE cohorts with respect to current corticosteroid use [33,34], prevalence of renal disease [1,30,33,34], mean organ damage index [1,34], and mean disease activity score [30,33], suggesting that race may not have had an important influence on our conclusions. Socioeconomic status, a factor influencing disease severity and prognosis in patients with SLE, was not assessed in the present study. Therefore, the results of our study may not be generalised to SLE cohorts of different socioeconomic background.

## Conclusion

The results of the present study emphasise that infection imposes a serious burden on patients with SLE. Although defects in the complement system have been suggested to be partially responsible for the high infection rate in patients with SLE, the results of our study suggest that deficiency of functional MBL activity does not play a role in the susceptibility to infections or major infections.

## Abbreviations

HRP = horseradish peroxidase; MASP = mannose-binding lectin-associated serine protease; MBL = mannose-binding lectin; SLE = systemic lupus erythematosus.

## Competing interests

The authors declare that they have no competing interests.

## Authors' contributions

IEMB collected clinical data, carried out the statistical analyses, and drafted the manuscript. DH supervised the assessment of functional MBL serum levels, C4 concentration, and MBL-induced C4 deposition and helped to draft the manuscript. MAS performed the MBL pathway activity enzyme-linked immunosorbent assay (ELISA) and helped to draft the manuscript. MHH performed the assessment of functional MBL serum levels, C4 concentration, and MBL-induced C4 deposition. BACD participated in the design of the study and reviewed the draft of the manuscript. MRD participated in the design of the study, supervised the performance of the MBL pathway activity ELISA, and reviewed the draft of the manuscript. AEV participated in the design of the study, contributed to the coordination of the study, supervised the statistical analyses, and reviewed the draft of the manuscript. All authors have read and approved the final manuscript.

## Figures and Tables

**Table 1:** Demographic, clinical, and therapy variables

Variables	All patients (*n *= 103)	Healthy controls
Demographic variables		
Female gender, percentage	91	
Caucasian race, percentage	77	
Age, years	41 ± 13	
Comorbidity		
Diabetes mellitus, percentage	4.9	
Clinical and laboratory variables		
Disease duration, years	6.8 ± 4	
SLEDAI	4.7 ± 4	
ECLAM	3.1 ± 1.6	
SLICC/ACR damage index	1.4 ± 1.9	
Previous biopsy proven lupus nephritis, percentage	31.1	
Creatinine clearance <70 ml/minute, percentage	18	
Proteinuria, percentage	13.6	
Leucopoenia ever, percentage	36.9	
Functional MBL serum level, median (range) μg/ml	1.4 (0.04–7.60)	1.1 (0.0–11.2)
Functional MBL serum level <0.05 μg/ml, percentage	13	14
MBL-induced C4 deposition on mannan, median (range) percentage	71 (1.1–399)	114 (3–494)
MBL-induced C4 deposition on mannan <10%, percentage	21	16
Functional activity of the MBL pathway, median (range) percentage	16 (0.1–119)	56 (0–133)
Functional activity of the MBL pathway <10%, percentage	43	28
C3 levels in serum, median (range) g/l	0.86 (0.06–1.70)	(0.88–2.01)
C4 levels in serum, median (range) g/l	0.13 (0.07–0.35)	0.21 (0.11–0.61)
Therapy variables		
Oral corticosteroids		
Ever user, percentage	81	
Current user, percentage	52	
Duration of corticosteroid use in ever users, months	63 ± 70	
Actual prednisone dose if >0 mg/day, mg	13 ± 12	
Intravenous methylprednisolone use ever, percentage	15	
Hydroxychloroquine use ever, percentage	87	
Azathioprine use ever, percentage	37	
Methotrexate use ever, percentage	14	
Cyclophosphamide (oral and/or intravenous) use ever, percentage	13	

Except where indicated otherwise, values are presented as the mean ± standard deviation. ECLAM, European consensus lupus activity measurement (range 0 to 10) [35]; MBL, mannose-binding lectin; SLEDAI, systemic lupus erythematosus disease activity index (range 0 to 105) [36]; SLICC/ACR, Systemic Lupus International Collaborating Clinics/American College of Rheumatology [37].

**Table 2:** Anatomic site of infections (*n *= 115) and isolated microorganisms in 103 patients with systemic lupus erythematosus

Anatomic site of infections (total number)	Microorganism
Skin and mucosa (33)	Herpes zoster (18), *Staphylococcus aureus *(1), *Candida albicans *(2), unidentified (12)
Lower respiratory tract (25)	*S. aureus *(2), *Klebsiella pneumoniae *(1), *Haemophilus influenzae *(1), Respiratory syncytial virus (1), unidentified (20)
Upper respiratory tract (16)	Herpes simplex (2), *Aspergillus fumigatus *(1), unidentified (13)
Genital (13)	*Chlamydia trachomatis *(3), *C. albicans *(3), *Trichomonas vaginalis *(1), *Gardnerella vaginalis *(1), Human papillomavirus (1), *Bacteroides fragilis *(1), unidentified (3)
Gastrointestinal tract (10)	*Salmonella typhi *(3), *Campylobacter jejuni *(2), *C. albicans *(2), unidentified (3)
Systemic (8)	*S. aureus *(5), *Streptococcus group A *(1), Cytomegalovirus and *C. albicans *(1), unidentified (1)
Musculoskeletal (4)	Osteomyelitis by *S. aureus *(1), septic arthritis of knee prosthesis by *S. aureus *(1), osteomyelitis by unidentified microorganism (2)
Upper urinary tract (3)	Unidentified (3)
Other (3)	*Pasteurella *(axillary abscess), *Streptococcus group A *(axillary and cervical abscess), unidentified (post-operative abdominal abscess)

Microorganisms were isolated in 57 (50%) of the 115 infections.

**Table 3:** Potential risk factors for the first major infection in 103 patients with SLE

	= 1 major infection (*n *= 23)	No major infection (*n *= 80)	*p *value
Laboratory variables			
Functional MBL serum level, μg/ml^a^	1.60 (0.04–7.00)	1.40 (0.04–7.60)	0.316
Functional MBL serum level <0.05 μg/ml	2/23 (9%)	11/80 (14%)	0.522
MBL-induced C4 deposition on mannan, percentage^a^	64.8 (3.4–400)	71.1 (1.1–400)	0.550
MBL-induced C4 deposition on mannan <10%	5/23 (22%)	16/80 (20%)	0.856
MBL pathway activity, percentage^a^	14.1 (0.1–111)	17.3 (0.1–119)	0.806
MBL pathway activity <10%	10/23 (44%)	34/80 (43%)	0.934
Clinical variables			
Disease duration at follow-up, years	12.5 ± 7.4	5.1 ± 5.7	0.0001
Previous SLE glomerulonephritis	10/23 (44%)	10/80 (10%)	0.001
Treatment variables	Use <3 months before the first major infection	Previous use ever since lupus diagnosis	
Oral corticosteroids	17/23 (74%)	41/80 (51%)	0.916
Intravenous methylprednisolone	5/23 (22%)	9/80 (11%)	0.204
Hydroxychloroquine	6/23 (26%)	70/80 (88%)	0.0001
Methotrexate	0/23 (0%)	9/80 (11%)	0.094
Azathioprine	8/23 (35%)	21/80 (26%)	0.424
Oral cyclophosphamide	1/23 (4%)	1/80 (1%)	0.373
Intravenous cyclophosphamide	4/23 (17%)	4/80 (5%)	0.065

Except where indicated otherwise, values are presented as the mean ± standard deviation. ^a^Values are presented as the median (range). MBL, mannose-binding lectin; SLE, systemic lupus erythematosus.

**Table 4:** Multivariate analysis of the first major infection (dependent variable) and clinical and therapy variables (independent variables)

Variables	Odds ratio	95% confidence interval	*p *value
Disease duration	1.18	1.07–1.31	0.001
Previous SLE glomerulonephritis	3.92	0.71–21.56	0.116
Hydroxychloroquine^a^	0.05	0.01–0.23	0.0001
Intravenous methylprednisolone^a^	2.18	0.36–13.13	0.392
Intravenous cyclophosphamide^a^	0.35	0.03–3.87	0.390

^a^Use within the last 3 months before the first major infection or previous use since lupus diagnosis if no major infection. SLE, systemic lupus erythematosus.
